# HiCuT: An efficient and low input method to identify protein-directed chromatin interactions

**DOI:** 10.1371/journal.pgen.1010121

**Published:** 2022-03-23

**Authors:** Satish Sati, Parker Jones, Hali S. Kim, Linda A. Zhou, Emmanuel Rapp-Reyes, Thomas H. Leung

**Affiliations:** 1 Department of Dermatology, University of Pennsylvania, Philadelphia, Pennsylvania, United States of America; 2 Department of Genetics, University of Pennsylvania, Philadelphia, Pennsylvania, United States of America; 3 Corporal Michael J. Crescenz Department of Veterans Affairs Medical Center, Philadelphia, Pennsylvania, United States of America; Netherlands Cancer Institute, NETHERLANDS

## Abstract

3D genome organization regulates gene expression, and disruption of these long-range (>20kB) DNA-protein interactions results in pathogenic phenotypes. Chromosome conformation methods in conjunction with chromatin immunoprecipitation were used to decipher protein-directed chromatin interactions. However, these methods required abundant starting material (>500,000 cells), sizable number of sequencing reads (>100 million reads), and elaborate data processing methods to reduce background noise, which limited their use in primary cells. Hi-C Coupled chromatin cleavage and Tagmentation (HiCuT) is a new transposase-assisted tagmentation method that generates high-resolution protein directed long-range chromatin interactions as efficiently as existing methods, HiChIP and ChIA-PET, despite using 100,000 cells (5-fold less) and 12 million sequencing reads (8-fold fewer). Moreover, HiCuT generates high resolution fragment libraries with low background signal that are easily interpreted with minimal computational processing. We used HiCuT in human primary skin cells to link previously identified single nucleotide polymorphisms (SNPs) in skin disease to candidate genes and to identify functionally relevant transcription factors in an unbiased manner. HiCuT broadens the capacity for genomic profiling in systems previously unmeasurable, including primary cells, human tissue samples, and rare cell populations, and may be a useful tool for all investigators studying human genetics and personalized epigenomics.

## Author summary

DNA is precisely packaged and organized within a nucleus to regulate gene expression. Altering this structure results in disease and developmental abnormalities. Current methods to probe 3D genome organization require a minimum of 500,000 cells, 100 million sequencing reads, and elaborate computational genomics skills. This limits general adoption and prevents use in small populations of cells, including primary tissues. We report a new method called Hi-C Coupled chromatin cleavage and Tagmentation (HiCuT). This method couples current 3D genome methods in conjunction with target specific tagmentation, an enzyme-assisted approach to cut and tag DNA. We benchmarked HiCuT against existing methods and found similar efficiency and specificity in assessing target specific 3D genome organization despite reducing assay requirements to 100,000 cells (5-fold less) and 12 million sequencing reads (8-fold fewer). Strikingly, HiCuT data exhibited reduced background noise and required minimal computational processing. Taken together, HiCuT broadens the capacity for 3D genome profiling in cell populations previously unmeasurable, including primary cells and human tissues, reduces sequencing costs, and lowers the need for computational expertise. HiCuT will benefit all investigators studying gene regulation and disease pathophysiology.

## Introduction

The structure and function relationship of the 3D genome organization remains a fundamental question in biology. 3D genome dynamics and functions during the cell cycle, development, gene transcription, and signalling have been studied in multiple cell types [1,2]. Disruption of the 3D genome results in distinct pathogenic phenotypes, including malformation of the skull and bones [3,4]. However, assessing 3D genome dynamics in human tissues, primary cells, and other rare cell populations has been limited.

Protein-DNA interactions are the basic unit of genome organization. Current methods to detect long-range chromatin interactions mediated by a specific protein factor include Hi-C sequencing coupled with chromatin immunoprecipitation-sequencing (ChIP-seq), chromatin interaction analysis by paired-end tag sequencing (ChIA-PET), proximity ligation-assisted ChIP-seq (PLAC-seq), and HiChIP [5–9]. These methods generally perform standard Hi-C followed by chromatin immunoprecipitation to capture DNA-protein complexes. Chromatin immunoprecipitation relies on non-specific chromatin fragmentation and immunoprecipitation which contributes to high background noise and a low signal-to-noise ratio. These methods require large amounts of starting material (500,000–100 million cells), sequencing reads (100–500 million reads per sample), and elaborate computational data processing algorithms to reduce background noise. A recently developed transposase-mediated tagmentation method (CUT&Tag) uses an enzyme-tethering strategy to improve capture of DNA-protein complexes, thereby increasing assay sensitivity and specificity and reducing the starting material required [10,11]. Here, we describe Hi-C Coupled chromatin cleavage and Tagmentation (HiCuT), a Hi-C tagmentation strategy that provides efficient and high-resolution protein directed long-range chromatin interactions from 100,000 cells and 12 million sequencing reads per sample. Relative to current methods, this assay reduces the starting material requirement by more than 5-fold, the sequencing depth requirement by 8-fold, and sample processing time by 50%.

## Results

**Fig 1:**
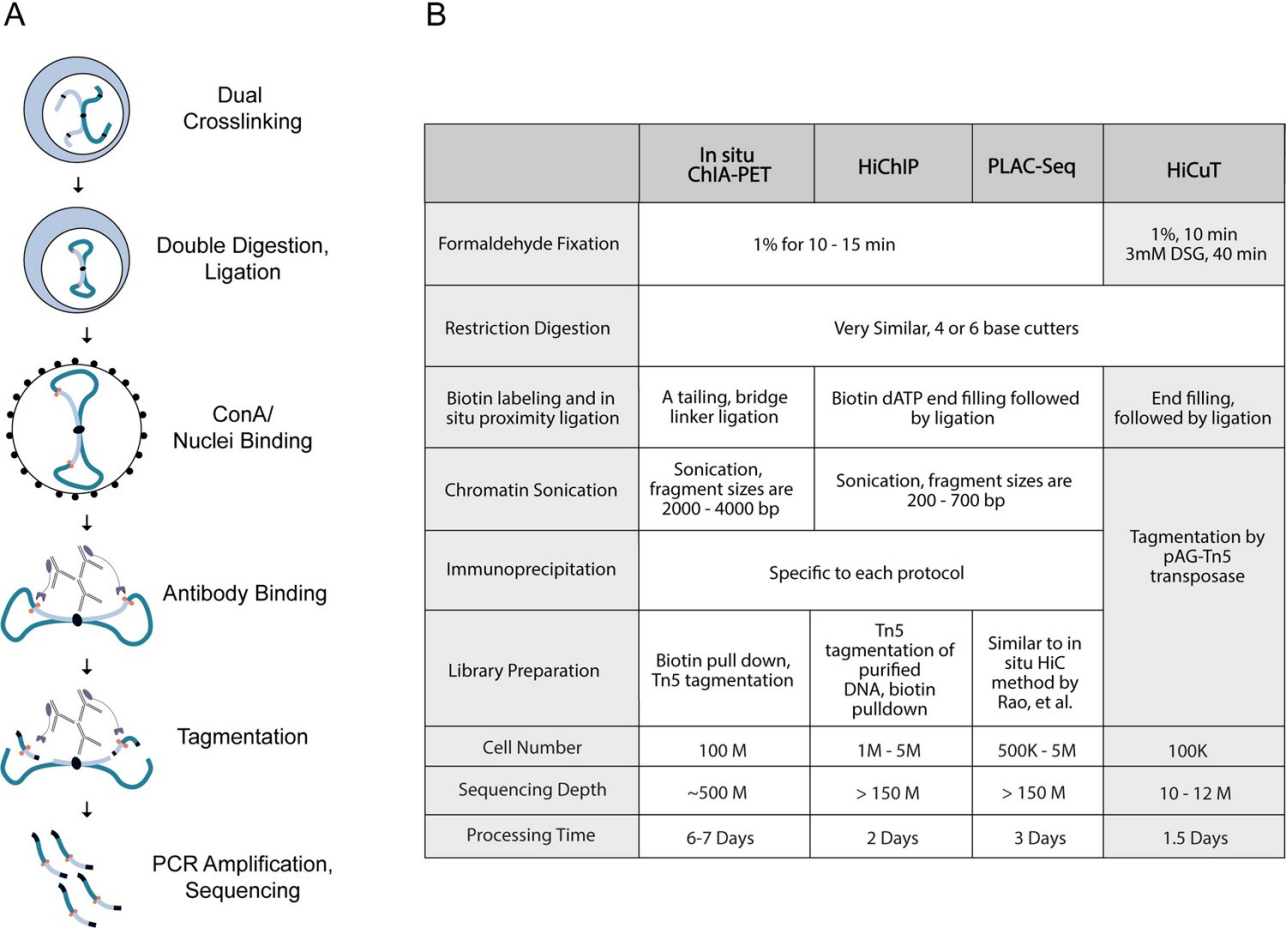
HiCuT identifies protein directed chromatin interactions in low cell numbers. (A) Method schematic. (B) Comparison of HiCuT protocol to existing methods.

Briefly, we dual crosslink cells with formaldehyde and disuccinimidyl glutarate (DSG). We perform Hi-C 3.0 with double restriction digestion with DdeI and DpnII enzymes [12,13]. Following proximity ligation, the nuclei are next conjugated with concanavalin-A beads and undergo antibody incubation to capture long-range interactions associated with our protein of interest. We then perform tagmentation using pAG-Tn5 transposases preloaded with sequencing adapters (Fig 1A and 1B). This tagmentation step captures protein-DNA complexes and simultaneously prepares DNA fragments for sequencing library amplification. After sequencing, we process our data using the HiC-Pro pipeline to identify informative unique paired-end tags [14].

We performed HiCuT using antibodies against CTCF, a well-established transcription factor that regulates 3D nuclear architecture, in 100,000 cells of the human B lymphocyte GM12878 cell line (S1 Table). HiCuT generated highly reproducible datasets with a Spearman correlation coefficient >0.7 between the biological replicates. We pooled three samples to create a 300,000 cells dataset for downstream analysis (S1A Fig). Mapped reads were strongly enriched at CTCF-binding sites identified by ENCODE GM12878 ChIP-Seq datasets, confirming the high specificity of the HiCuT assay towards profiling DNA binding factor occupancy (S1B, S1C, and S1G Fig) [15]. To assess the specificity of protein mediated interactions detected using HiCuT, we processed HiCuT data using the HiC-Pro pipeline. We retained long-range chromatin interactions between 20kb to 2Mb, with at least one end overlapping with a known published ENCODE CTCF ChIP-seq peak (S2 Fig and S1 Table).

**Fig 2:**
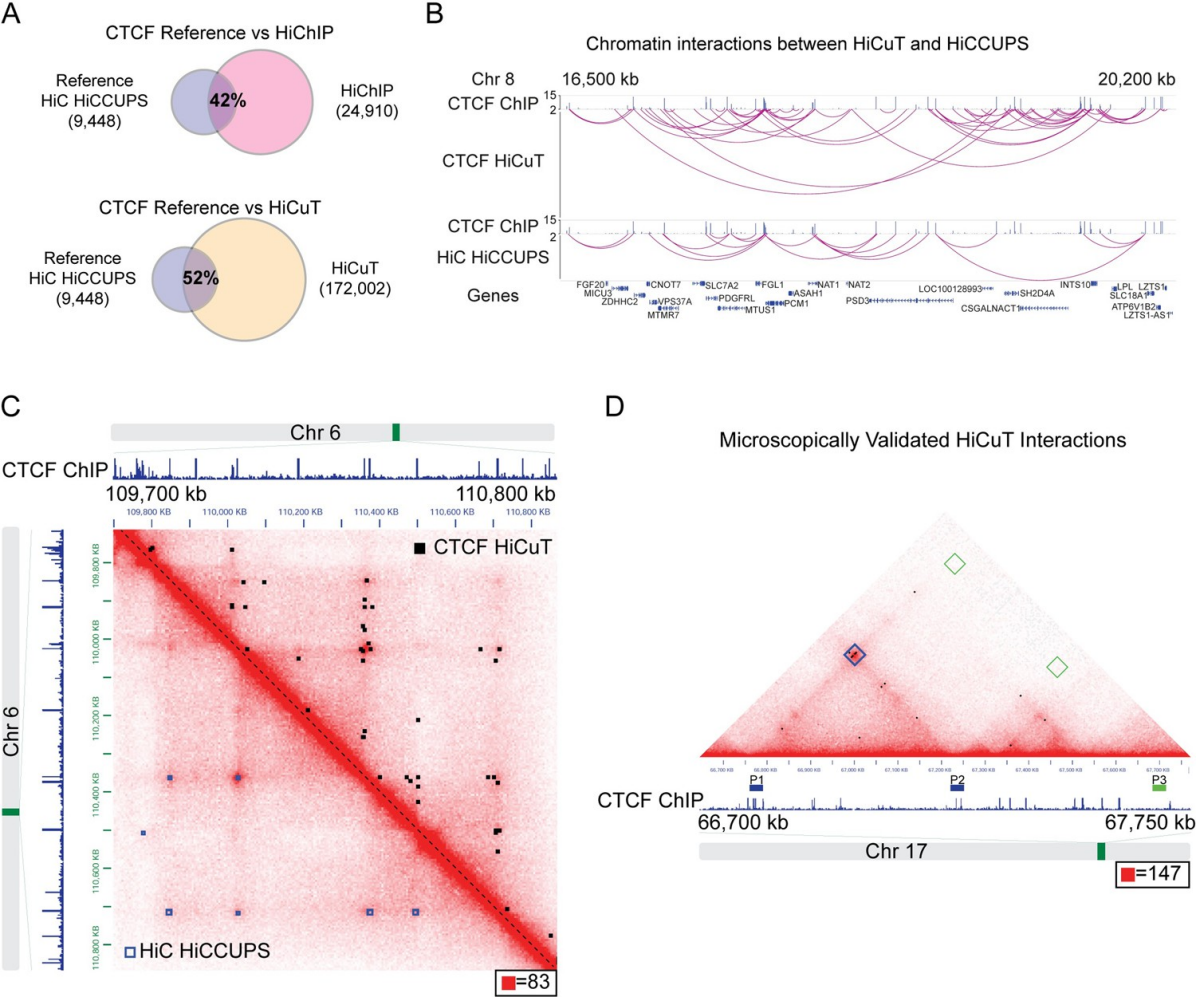
HiCuT identifies CTCF mediated long-range interactions in GM12878 cells. (A) Comparison of shared long-range chromatin interactions between Hi-C HICCUPS loop reference data set, GM12878 CTCF HiChIP loops, and GM12878 CTCF HiCuT interactions. The number of interactions for each dataset are displayed. (B) WashU Epigenome Browser view of chromatin interactions in GM12878 cells. CTCF ChIP tracks are from ENCODE (GSM733752) followed by chromatin interactions from CTCF HiCuT or Hi-C loops identified by HiCCUPS [15]. Gene names listed below. Chr, Chromosome. (C) GM12878 Hi-C contact map superimposed with the CTCF HiCuT interactions (upper right of map, black boxes) and Hi-C loops identified by HiCCUPS (lower left of map, open blue boxes). CTCF ChIP tracks are from ENCODE (GSM822312) [15]. Maximum intensity is indicated in the lower right of each panel. (D) GM12878 Hi-C contact map with published location of *in situ* DNA FISH probes (P1 to P3) previously used to verify a chromatin loop on the chromosome 17 (blue box) [13]. CTCF Hi-CuT interactions (black boxes) are superimposed onto this map. CTCF ChIP tracks are from ENCODE (GSM822312) [13,15]. Maximum intensity is indicated in the lower right of each panel.

Given the low cell number and decreased sequencing depth, we expect HiCuT to largely detect high frequency chromatin interactions. We compared interactions obtained from HiCuT to published loop calls from a validated and heavily referenced Hi-C dataset generated from ~125 million GM12878 cells and ~ 6.5 billion paired end reads [13]. Matched loops shared both loop anchors. Our HiCuT data set captured 52% of all loops called from Hi-C data [13]. This overlap is comparable to the 42% overlap observed between the Hi-C loops and a published 2 million cell HiChIP dataset, despite the HiChIP dataset being generated from ~7-fold more cells and 18-fold more sequencing reads [16] (Figs 2A and 2B and S1D, and S1 and S2 Tables). We visualized our HiCuT data on juicebox and overlaid the interactions onto a Hi-C map (Figs 2C, S1E, and S3A–S3C) [13,15,17,18]. In the juicebox panel, top-half of the map displayed the HiCuT data, where the black boxes identify long-range loops, and the bottom-half of the map displayed long-range loops called by the published HiCCUPS method (open blue boxes) [13]. The captured loops strikingly mirror each other. Thus, HiCuT captured most, if not all, of the major identified Hi-C loops (S1E Fig). Next, we used aggregate peak analysis (APA) to quantify the aggregate enrichment of the entire set. We aggregated HiCuT and HiChIP interaction counts over pairwise combination of CTCF-ChIP peaks falling within a 5kb to 1Mb distance interval. Compared to HiChIP, CTCF HiCuT datasets generated higher center enrichment and APA scores (S1F Fig). Importantly, 80% of HiCuT interactions colocalize with a validated CTCF ChIP-Seq peak, indicating that the HiCuT interactions are highly specific (S1 Table).

CTCF frequently colocalizes with the cohesin protein complex, and a SMC1a cohesin HiChIP dataset generated from 25 million GM12878 cells also had a 38% overlap against the Hi-C data set, which was similar to our 52% CTCF HiCuT overlap (S3D and S3E and S4A and S4B Figs) [7]. Finally, CTCF HiCuT data appropriately identified published in-situ HiC and DNA fluorescence in situ hybridization (DNA-FISH) validated long-range CTCF-mediated loops (Figs 2D, S3F–S3I, and S4C) [13]. Taken together, CTCF HiCuT identifies long-range interactions as effectively as existing methods. Importantly, compared to a full loop calling pipeline like HiCCUPS, HiCuT requires only minimum processing of interactions to reliably detect high frequency chromatin contacts.

**Fig 3:**
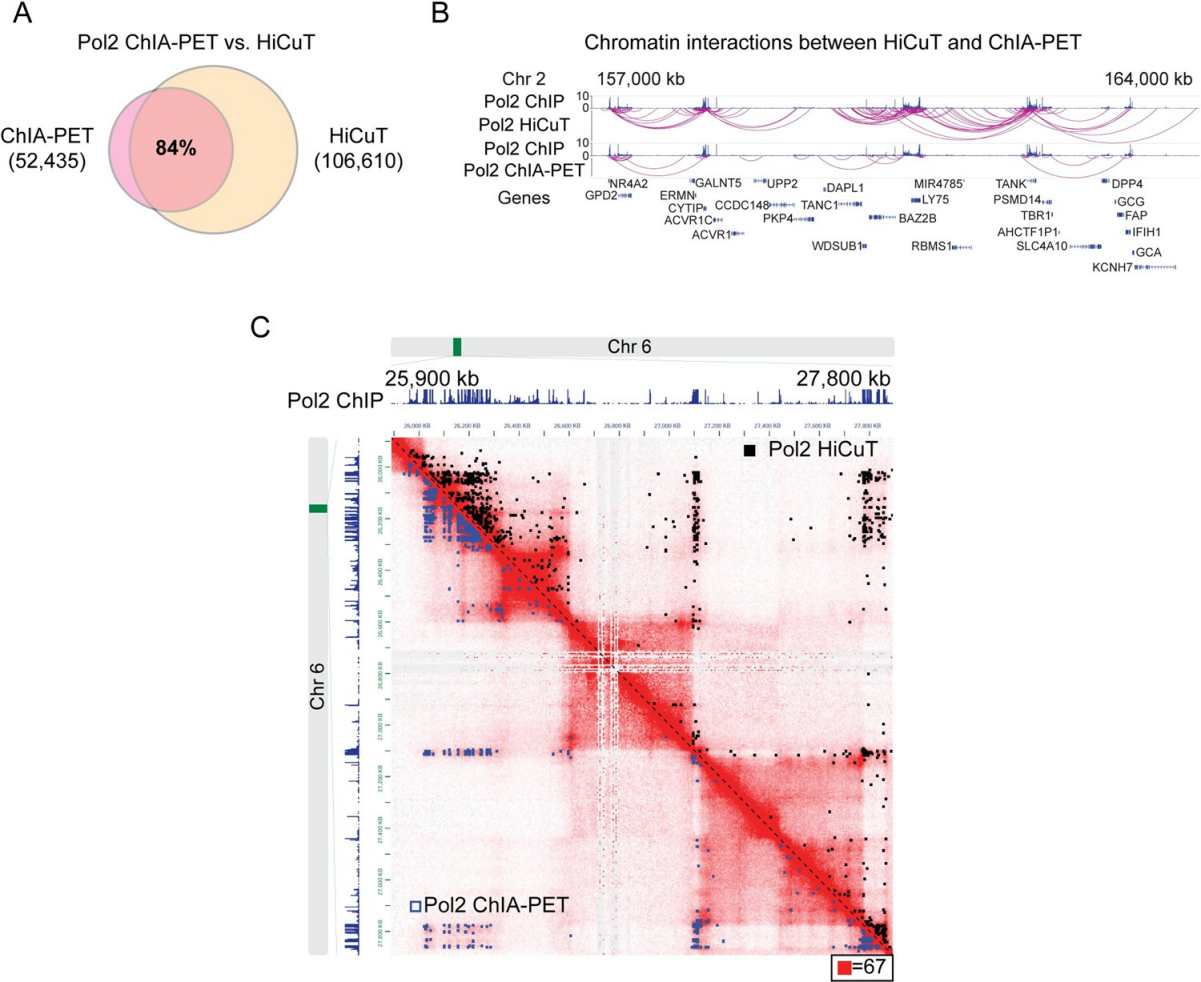
HiCuT identifies RNA Pol II mediated long-range interactions in GM12878 cells. (A) Comparison of shared long-range chromatin interactions between GM12878 RNA polymerase 2 (Pol2) HiCuT and ChIA-PET loops. The number of interactions for each dataset are displayed. (B) WashU Epigenome Browser view of chromatin interactions in GM12878 cells. The Pol2 ChIP tracks are from ENCODE GM12878 cells (GSM935386) followed by chromatin interactions identified by HiCuT or ChIA-PET. Chr, Chromosome [15,17]. (C) RNAPol2 Hi-C contact map at 5 kb resolution superimposed with RNAPol2 HiCuT (upper right, black boxes) and RNAPol2 ChIA-PET interactions (lower left, blue boxes) [8,15]. Maximum intensity is indicated in the lower right of each panel.

We extended our analysis and performed HiCuT using antibodies against RNA Polymerase 2 (Pol2) in GM12878 cells. HiCuT generated highly reproducible datasets and mapped reads were strongly enriched at Pol2-binding sites identified by ENCODE GM12878 ChIP-Seq datasets (S5A–S5D Fig). HiCuT identified ~106,000 long-range interactions (Fig 3A and S1 Table). We compared our dataset to a published Pol2 chromatin interaction analysis by paired-end tag sequencing (ChIA-PET) dataset generated from 100 million GM12878 cells [8]. Our 300,000 cell HiCuT dataset captured 84% of the identified interactions, despite using 325-fold less starting material (100 million cells in ChiA-PET) and ~40-fold fewer sequencing reads (Fig 3A and S2 Table). We visualized both datasets onto a Hi-C map and WashU browser, and most, if not all, of the major interactions are detected by the HiCuT method (Figs 3B and 3C, S3J, and S5E) [8]. Compared to ChIA-PET, Pol2 HiCuT datasets generated higher center enrichment and APA scores (S5D Fig). Moreover, 63% of HiCuT interactions also colocalized with a validated Pol2 ChIP-Seq peak (S1 Table). Thus, HiCuT robustly captures long-range chromatin interactions for multiple proteins.

**Fig 4:**
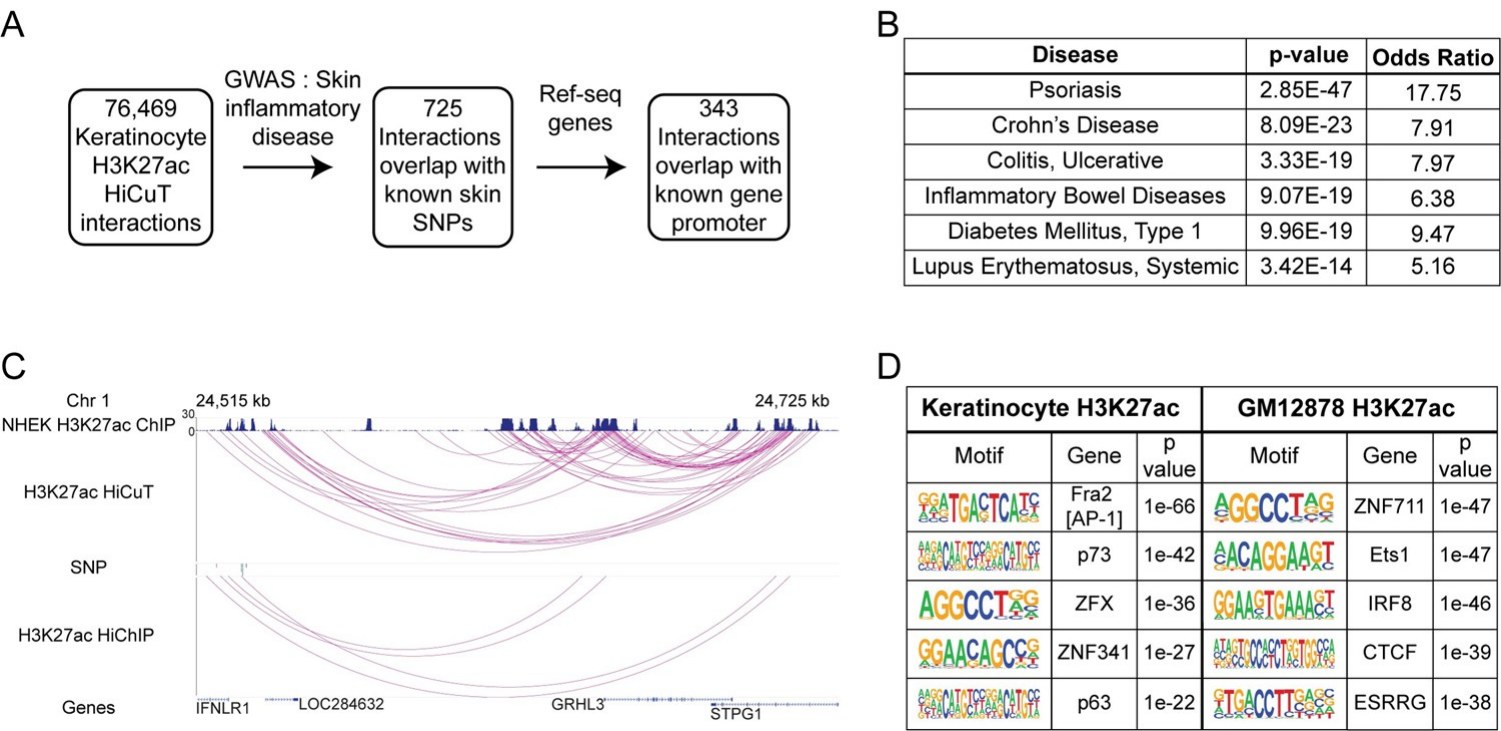
HiCuT identifies long-range promoter-enhancer interactions in primary human keratinocytes. (A) Identifying candidate genes associated with inflammatory skin disease specific single nucleotide polymorphisms (SNPs). (B) Candidate genes analysed by gene ontogeny using EnrichR [19]. (C) WashU epigenome browser view of chromatin interactions in primary human keratinocytes. The H3K27ac ChIP tracks from are from ENCODE NHEK cells (GSM733674) followed by chromatin interactions identified by HiCuT or HiChIP [15,17]. Chr, Chromosome. (D) Top transcription factor binding motifs identified by Homer in of H3K27ac HiCuT anchor regions from primary keratinocytes and GM12878 cells [37].

Existing methods to detect long-range interactions have limited use in human primary cells, where input cell number is restricted. To determine the efficacy of HiCuT to overcome this limitation, we performed HiCuT in 100,000 primary human keratinocytes using antibodies against histone 3 lysine 27 acetylation (H3K27ac), a well-known epigenetic mark of active enhancers (S6A–S6D Fig). In our pooled 300,000 cell dataset, HiCuT identified ~76,000 long-range interactions (Fig 4A and S1 Table). We compared these interactions with known single nucleotide polymorphisms (SNPs) identified in GWAS studies on human inflammatory skin diseases (NHGRI-EBI catalog, EFO_0000676). 725 interactions overlapped with known SNPs, and 343 of those interactions had one anchor originating from a gene promoter (Fig 4A and S3 Table). These candidate genes were further analysed by gene ontogeny using EnrichR, and two of the top 6 hits were related to inflammatory skin diseases, with psoriasis being the top hit (Fig 4B) [15,17,19]. HiCuT also appropriately captured an established and validated locus containing multiple long-range SNP-gene interactions (Fig 4C) [20]. Thus, HiCuT linked previously identified SNPs to potential candidate genes (S6E Fig). Finally, we computationally interrogated all identified anchor sequences for over-represented transcription factor binding sites, and the top 4 hits of inferred transcription factors include: p63, Fra-2, p73, and ZFX, all well-established mediators of keratinocyte function (Fig 4D) [21–24]. As a negative control, similar analysis of a HiCuT H3K27ac dataset from GM12878 cells revealed a different non-overlapping set of inferred transcription factors (Figs 4D and S6F).

## Discussion

In this study, we generated high-confidence protein-directed chromatin interaction profiles from 100,000 cells using antibodies against CTCF, RNAPol2, and the active enhancer mark H3K27ac. We found comparable efficiency in detection of long-range interactions when we benchmarked HiCuT against published datasets that used recommended cell numbers of existing techniques. As expected, HiCuT produced fewer unique valid interactions compared to existing methods, because we start with fewer cells and sequencing reads (S1 Table). However, the percentage of unique valid interactions and the cis:trans interaction ratios remain in-line with existing methods.

One of the most striking aspects of HiCuT is how high frequency protein mediated long range interactions are easily obtained and interpretable with minimal computational processing, as there is extremely limited nonspecific background signal (as illustrated in S2 Fig). In fact, 60–80% of mapped long-range HiCuT interactions fall specifically within known ChIP peak sites, highlighting the specificity of this assay (S1 Table). This reduced background noise allows HiCuT datasets to forgo the use of loop calling programs or filtering pipelines required by existing assays, and this also reduces the number of sequencing reads required per sample (S1 Table and S2 Fig). Several aspects of our protocol highlight how this is possible: 1) Tagmentation generates highly specific and small amounts of DNA fragments. We achieve high resolution for loop origins. 2) We omit the Hi-C biotin pulldown step to capture more DNA, which minimizes PCR bias during the amplification step of sequencing library generation. Omitting this step also did not introduce additional noise as the percentage of long-range reads compared to total reads remains similar between HiCuT and HiChIP (S1 Table). 3) We simply use the distance threshold on the HiC-Pro pipeline output to select for long-range interactions. These interactions remain “unprocessed” in contrast to loop filtering algorithms, which may differ substantially between each other based on parameter definitions and strategies. Individual loop filtering algorithms analysing the same dataset identify different numbers of loops [25]. 4) We utilize the Hi-C 3.0 protocol which included the use of double fixation and double restriction enzyme digest and shown to be 2-fold more sensitive in detecting long range chromatin loops than the Hi-C 2.0 protocol, which all existing methods used [12,26]. With these limited steps, we enrich for long range interactions at a similar level of efficiency to existing methods despite less starting material, fewer sequencing reads, and minimal computational processing. Finally, HiCuT simultaneously generates high-quality DNA binding data. ~80% of HiCuT CTCF interactions originate from a known protein-binding site, and HiCuT CTCF peaks match ~30% of published ChIP-Seq and CUT&RUN peaks (S1G Fig and S1 Table). This offers investigators profiling long range protein directed interactions added information about DNA protein occupancy without additional investment.

In past few years, successful attempts were made to lower the amount of starting material for Hi-C based assays [27,28]. In particular, single cell Hi-C (scHi-C) detects long-range interactions from single cells [29,30]. One weakness of scHi-C, shared by all single-cell methods, is that the depth of potential interactions detected is extremely limited. Protein directed population methods permit a deeper characterization of long-range interactions. Tagmentation has enabled researchers to generate ChIP-Seq comparable profiles more efficiently and from smaller cell populations [31,32]. Our individual 100,000 cell CTCF HiCuT replicates captured on average ~45% of the combined 3 sample dataset (S1D Fig). Future studies are needed to further scale down this method to lower cell numbers or even single cells.

HiCuT and other protein-directed 3C assays frequently detect more long-range interactions compared to traditional Hi-C. This difference in interactions may be due to insufficient sequencing depth of the Hi-C assay or protein-directed methods offer additional fidelity due to the enrichment of specific binding sites. One limitation of all protein-directed assays is that we cannot compare the capture frequencies of enriched regions to non-bound or non-enriched sequences. While we cannot rule out the possibility that these additional interactions are background noise, we note that HiCuT does not require loop calling algorithms, and the majority of the additional interactions fall in verified protein binding sites (S1 Table). Thus, the functional relevance of these additional interactions will need further experimental validation.

In conclusion, we present HiCuT, a rapid, low input, and cost-effective method to generate genome-wide chromatin interaction maps from 100,000 cells and 12 million reads per sample. In addition to assessing 3D genome architecture, potential applications for HiCuT in primary human tissues include functionally linking previously identified SNPs to disease causing genes and the unbiased identification of functionally relevant transcription factors. The use of HiCuT with H3K27ac will allow detection of enhancer promoter interactions without knowing a protein factor a priori. HiCuT bypasses limitations of existing chromatin interacting methods and broadens the capacity for genomic profiling in systems previously unmeasurable, including primary cells, human tissue samples, and rare cell populations.

## Materials and methods

### Cell culture and antibodies used

We used two different cell types, surface adherent primary keratinocytes and surface non-adherent floating GM12878 cells. GM12878 cells were provided by Dr. A. Raj (University of Pennsylvania). GM12878 cells were cultured in RPMI (Thermo-Fisher Scientific, Cat.No: 11875–085), supplemented with 10% fetal bovine serum and 50U of penicillin and streptomycin (Thermo-Fisher Scientific, Cat.N: 15070–063). Primary keratinocytes were provided by University of Pennsylvania Department of Dermatology, Skin Biology and Diseases Resource-based Center (SBDRC). Cells were grown in supplemented 50:50 keratinocyte media, a 50:50 mixture of keratinocytes-SFM (Thermo Scientific) and Medium 154 (Thermo Scientific). Cells were grown at 37°C and 5% CO2.

### Cell lysis and nuclei fixation

We fixed 100,000 cells in 0.5 mL of freshly made 1% formaldehyde solution at room temperature for 10 minutes. To quench the formaldehyde, we added glycine to a final concentration of 200 mM for 5 minutes at room temperature and then 15 minutes on ice. Cells were washed once with 0.05% BSA in PBS and spun down at 2,000 g for 5 minutes. We fixed cells for a second time with 3mM DSG (final concentration) in 500 μL PBS, at room temperature for 40 minutes, on rotation. We added glycine at a final concentration of 0.4 M for 5 minutes. Cells were washed once with 0.05% BSA in PBS and spun down at 2,000 g for 5 minutes. We resuspended cells in Hi-C lysis buffer (10mM Tris-HCl pH8.0, 10mM NaCl, 0.2% Igepal CA630, 1X protease inhibitor) and incubated them on ice for 30 minutes. We spun down the cells at 2,500 g for 5 minutes and washed the nuclei once with NEBuffer 3.1.

### *In situ* contact generation

*In situ* contacts were generated according to the *in situ* Hi-C protocol with minor modifications. We resuspended nuclei in 161 μL of 1x NEBuffer 3.1 and permeabilized them by adding 19 μL of 1% SDS and incubating the mixture for 10 minutes at 65°C without shaking. Immediately afterwards, we placed the tube on ice. We quenched the SDS by adding 21.5 μL of 10% Triton X-100 and incubating the samples at 37°C for 15 minutes with shaking at 900 rpm. Next, we added 20 μL of 10U/μL DdeI, 4 μL of 50U/μL DpnII, and 2 μL of 1x NEBuffer 3.1 and mixed gently by pipetting. The mixture was incubated for 3 hours or overnight at 37°C on a thermomixer at 900 rpm, in 30 seconds on, 4 minutes off mode. After digestion, we inactivated the enzymes at 65°C for 20 minutes with no shaking. To fill in restriction fragment overhangs, we added 35 μL of end-filling master mix: 18.75 μL of 0.4 mM dATP; 0.75 μL of dTTP, dGTP, and dCTP at 10 mM each; and 5 μL of DNA polymerase I (NEB, M0210). We rotated the samples for 2–3 hours at 37°C. We ligated the DNA fragments by adding 332.5 μL of ligation master mix containing: 60 μL of 10X NEB T4 DNA ligase buffer (NEB, B0202), 50 μL of 10% Triton X-100, 6 μL of 10 mg/mL BSA, 2.5 μL of 400 U/μL T4 DNA Ligase (NEB, M0202), and 214 μL of water. Samples were rotated end over end at room temperature for 2–3 hours. The nuclei were pelleted and washed once with 200 μL of exchange buffer (20 mM HEPES-KOH pH 7.9, 10 mM KCl, 0.1% Triton X-100, 20% Glycerol, 0.5 mM Spermidine, 1x EDTA-free Protease Inhibitor). The proximity ligated nuclei were resuspended in 100 μL of exchange buffer.

### Chromatin cleavage and tagmentation

We washed 10 μL of Concanavalin-A Beads two times with 100 μL of bead activation buffer (20 mM HEPES, pH 7.9, 10 mM KCl, 1 mM CaCl2, 1 mM MnCl2) and resuspended them in 100 μL of cold bead activation buffer. Beads were added to 25ul of the mixture, and the mixture was incubated at room temperature for 10 minutes. The tubes were placed on a magnetic stand, and the supernatant was removed.

We added 50 μL of cold antibody buffer (20 mM HEPES pH 7.5, 150 mM NaCl, 0.5 mM Spermidine, 1x EDTA-free Protease Inhibitor, 0.01% Digitonin, 2 mM EDTA) and 1 ug of the appropriate primary antibody (RNA Polymerase II (Cell Signaling Technology: Cat. 2629), H3K27ac (Active Motif: Cat. 39133), and CTCF (Cell Signaling Technology: Cat. 3418). Samples were incubated for 2 hours at room temperature or overnight at 4°C on a rotating platform. Next, we placed the samples on a magnetic stand and removed the supernatant. 50 μL of cold low-salt digitonin buffer (20 mM HEPES pH 7.5, 150 mM NaCl, 0.5 mM Spermidine, 1x EDTA-free Protease Inhibitor, 0.01% Digitonin) and 0.25 ug of secondary antibody were added (Anti-Rabbit (EpiCypher: Cat. 13–0047) and Anti-Mouse (EpiCypher: Cat. 13–0048)). Samples were incubated at room temperature for 30 minutes and then washed twice with 200 μL of cold low salt digitonin buffer. Next, 50 μL of ice-cold high salt digitonin buffer (20 mM HEPES pH 7.5, 300 mM NaCl, 0.5 mM Spermidine, 1x EDTA-free Protease Inhibitor, 0.01% Digitonin) and 2.5 μL of CUTANA pAG-Tn5 (20x stock from EpiCypher) were added, and samples were incubated at room temperature for 1 hour. They were washed twice with 200 μL cold digitonin high salt digitonin buffer. Next, samples were resuspended in 50 μL of cold tagmentation buffer (20 mM HEPES pH 7.5, 300 mM NaCl, 0.5 mM Spermidine, 1x EDTA-free Protease Inhibitor, 10 mM MgCl2) and incubated at 37°C for 1 hour. We removed the supernatant and resuspended the samples in 50 μL of Release Buffer (10 mM TAPS pH 8.5, 0.2 mM EDTA 0.5% SDS, 22.5mM EDTA and 1uL of 20mg/mL Proteinase K). Samples were incubated at 58°C for 1 hour and 68°C for 2 hours in a thermocycler. After incubation, supernatant was collected and purified with Zymo Research ChIP DNA Clean & Concentrator protocol (Cat No. D5210), as per manufacturer’s recommendation. We eluted the PCR-ready HiCuT libraries in 21 μL volume.

### Library amplification

We added to each sample, 2 μL of universal i5 primer, 10 μM of barcoded i7 primers (EpiCypher), and 25 μL of CUTANA High Fidelity 2x PCR Master Mix (EpiCypher). Primer sequences are listed in S4 Table. The following PCR settings were used: 58°C for 5 min, 72°C for 5 min, 98°C for 45 sec, then cycle at 98°C for 15 sec, 60°C for 10 sec, and 72°C for 1 min. Samples were amplified for 18 cycles. Size selection was performed using Ampure XP beads following the manufacturer’s recommendation. Libraries were eluted in 15 μL of elution buffer (Qiagen) and quantified using both a Qubit fluorometer and qPCR against Illumina primers. Libraries were sequenced in 75 bp paired-end sequencing format (Illumina NextSeq). Processing time for this protocol is 1.5 days.

### ChIP-Seq analysis

Fastq files were aligned to the hg19 reference genome. PCR duplicates were removed using Samtools and normalized genome coverage tracks were generated from uniquely mapped reads (mapq > 30) using deepTools2. Datasets were then processed using the MACS2 with default parameters, and peaks were called in both individual replicates and in the pooled dataset. We used bedtools intersect to identify common peaks between replicates [33,34]. We used publicly available ChIP-seq datasets in our analysis: GM12878 CTCF: GSM822312, GSM733752; GM12878 SMC3: GSM935376; GM12878 RNA Polymerase 2: GSM935386; GM12878 H3K27ac: GSM733771; NHEK H3K27ac: GSM733674. The CTCF CUT&RUN datasets (4DNES6GVE8XZ) in our analysis were taken from 4DN Network data portal (https://data.4dnucleome.org/) [35].

### HiCuT data analysis

Paired-end reads were aligned independently to the hg19 human genome using bowtie2 (global parameters:—very-sensitive–L 30 –score-min L,-0.6,-0.2 –end-to-end—reorder; local parameters:—very-sensitive–L 20 –score-min L,-0.6,-0.2 –end-to-end—reorder) through the HiC-Pro software [14]. The valid pair file was used for downstream analysis. Trans and < 1kb interactions were filtered out. For comparative analyses, we identified chromatin interactions that fall between a 20Kb to 2Mb window that have at least one anchor of the interacting pair falling in the ChIP peak region. Following the ChIP peak overlap and distance thresholding, we consider each valid pair as an individual interaction. If two overlapping valid pairs were present, we considered them as two interaction points. The QC metrics and the final interactions are provided in S1 and S2 Table.

For comparison to our HiCuT datasets, we used published loop coordinates from the following sources: GM12878 HiChIP Smc1a: GSE80820; GM12878 HiChIP CTCF: GSE115524; GM12878 HiC HICCUP loops: GSE63525; and GM12878 ChIA-PET RNA Polymerase2 loops: GSM1872887. The GM12878 ChIA-PET RNA Polymerase2 interacting pairs for APA analysis were taken from 4DN Data portal (4DNESZ25M0ZV) [35].

### Scatterplots and correlations

The fastq files from HiCuT samples were aligned to the hg19 reference genome using bowtie2. Following conversion from.sam to.bam format, the.bam files were processed using bamcompare from deepTools2.0 with default settings. The output was used to calculate Spearman correlation between replicates using plotCorrelation tool from deepTools2.0 [34].

### Visualization of HiCuT interactions

The HiCuT interactions and ChIP-Seq peaks from ENCODE were visualised using Juicebox and WashU Epigenome legacy browser [18,36]. The HiC maps in the Juicebox images were taken from the Juicebox archive for respective cell lines.

### Motif analysis

We identified H3K27ac HiCuT anchors which do not fall within +/- 2500 bp of known Ref-seq promoters or transcription start sites (from UCSC table browser). Since HiCuT interactions are 1 bp in size, the filtered anchors were extended 100 bp on each side [37]. We used HOMER Motif Analysis software (http://homer.ucsd.edu/homer/motif/).

### SNP analysis

The SNP coordinates were taken from GWAS dataset (Id: EFO_0000676), downloaded from NHGRI-EBI Catalog of human genome-wide association studies (https://www.ebi.ac.uk/gwas/home). Since our primary keratinocyte H3K27ac HiCuT interactions are 1 bp in size, we applied a 5kb window using bedtools v2.30.0 window function to identify HiCuT interactions falling in the vicinity of a SNP locus. The SNP matched interactions were then mapped to +/- 2500 bp of known Ref-seq promoters (from UCSC table browser). We identified 343 unique genes (S3 Table).

### Aggregate peak analysis (APA)

APA plots were generated for the following sets of interactions and loci:

1. CTCF HiCuT interactions at GM12878 CTCF ChIP-seq peaks
2. Pol2 HiCuT interactions at GM12878 Pol2 ChIP-seq peaks
3. H3K27ac HiCuT interactions at Keratinocyte H3K27ac ChIP-seq peaks
4. CTCF HiChIP interactions at GM12878 CTCF ChIP-seq peaks
5. GM12878 HiC interactions at GM12878 CTCF ChIP-seq peaks
6. Pol2 ChIA-PET interactions at GM12878 Pol2 ChIP-seq peaks

For each set, loci over which to plot interactions were determined by taking all pairwise combinations of peaks which were at least 5,000 bp away but less than 1 MB away were created. Out of this list, 200,000 random loci pairs were chosen per APA plot. APA plots were generating using the apa function of the software package juicer (v 1.6) [38]. Data normalized using Knight-Ruiz balancing was plotted.

## Supporting information

S1 Fig Supporting data for GM12878 CTCF HiCuT. (A) Scatter Plot correlation of aligned reads from HiCuT replicates. Spearman r is indicated. (B) Genome browser snapshot showing GM12878 CTCF HiCuT tracks (red), CTCF CUT&RUN Tracks from 4DN Network (green, 4DNES6GVE8XZ) and CTCF ChIP-Seq tracks from ENCODE (blue, GSM733752) [15,17,35]. (C) Violin plots of mapped reads from CTCF HiCuT datasets at CTCF ChIP peaks and random sites. (D) Comparison of HiCuT shared long-range chromatin interactions between Hi-C HICCUPS loop reference data set and HiCuT replicates. (E) In situ GM12878 Hi-C contact map of two regions at 5 kb resolution, superimposed with HiCuT interactions (top panels, upper right, black boxes), GM12878 Hi-C HiCCUPS loops (all panels, lower left, open blue boxes) and GM12878 CTCF HiChIP loops (lower panels, upper right, black boxes). Maximum intensity is indicated in the lower right of each panel. (F) APA plots from CTCF HiCuT, CTCF HiChIP and GM12878 HiC, around pairs of CTCF-binding sites from GM12878 cells. (G) Comparison of CTCF peaks obtained from GM12878 cells using HiCuT, ChIP-Seq (ENCODE GSM733752) and CUT&RUN (4DN network 4DNES6GVE8XZ). Number of identified peaks are shown.
(TIF)

S2 Fig Identifying long-range interactions using HiCuT and existing methods. (A) and (B). Two different chromosome regions are displayed. The left panel displays raw unique interactions for HiCuT, HiChIP, and Hi-C in GM12878 cells. The HiC interactions are taken from WashU browser (25 Kb bin, KR Norm). The middle panel displays filtered unique interactions for HiCuT and HiChIP. Captured interactions were between 20Kb– 2Mb in length, with at least one anchor overlapping with a known CTCF ChIP-seq peak. The right panel displays final long-range interactions after looping calling programs were performed for HiChIP and HiC datasets. HiCuT did not require additional filtering. ChIP-Seq tracks are obtained from ENCODE GM12878 dataset (GSM733752), and gene names are listed below [15,17]. Chr, Chromosome
(TIF)

S3 Fig Raw Hi-C plots from juicer. In situ GM12878 Hi-C data maps generated from juicer [13,18,38]. No datasets are superimposed. The corresponding figure panel is indicated at the top of each map.
(TIF)

S4 Fig Validation of GM12878 CTCF HiCuT interactions. (A) Comparison of shared long-range chromatin interactions between Hi-C reference data set, GM12878 CTCF HiCuT, and GM12878 SMC1a HiChIP. The number of interactions for each dataset are displayed [7]. (B) GM12878 Hi-C contact maps at two different loci. Hi-C dataset at 5kb resolution superimposed with HiCuT interactions (top panels, upper right, black boxes), GM12878 Hi-C HiCCUPS loops (all panels, lower left, open blue boxes) and GM12878 SMC1a HiChIP loops (lower panels, upper right, black boxes) (GEO GSE80820). Maximum intensity is indicated in the lower right of each panel [7]. (C) HiCuT captures previously published microscopically validated loops [13]. GM12878 Hi-C contact map with superimposed location of different DNA FISH probes. The blue probes (P1 and P2) were shown to interact in a DNA FISH experiment (blue rectangle), and HiCuT detected this interaction [13]. The green boxes represent non interacting regions between FISH probes (P1 to P3, green boxes). GM12878 CTCF Hi-CuT interactions (black boxes) superimposed on the in situ Hi-C map. Maximum intensity is indicated in the lower right of each panel.
(TIF)

S5 Fig Supporting data for GM12878 RNA polymerase 2 HiCuT. (A) Scatter plot correlation of aligned reads from HiCuT replicates. Spearman r is indicated. (B) Genome browser snapshot showing GM12878 RNA polymerase 2 HiCuT tracks (red) and RNA polymerase 2 ChIP-Seq tracks from ENCODE (blue, GSM935386) [15,17]. (C) Violin plot of mapped reads from HiCuT datasets at RNA polymerase 2 ChIP peaks and random sites. (D) APA plots for Pol2 HiCuT and Pol2 ChIA-PET around pairs of Pol2-binding sites from GM12878 cells. (E) WashU epigenome browser view of three different genomic regions highlighting protein-directed chromatin interactions. The RNA polymerase 2 ChIP tracks are from ENCODE GM12878 cells (GSM935386), followed by chromatin interactions identified by HiCuT and ChIA-PET assays [15,17]. Chr, Chromosome.
(TIF)

S6 Fig Supporting data for primary keratinocyte H3K27ac HiCuT. (A) Scatter plot correlation of aligned reads from HiCuT replicates. Spearman r is indicated. (B) Violin plot of mapped reads from HiCuT datasets at H3K27ac ChIP peaks and random sites. (C) Genome browser snapshot showing primary keratinocyte H3K27ac HiCuT tracks (red) and NHEK H3K27ac ChIP-Seq tracks ENCODE (GSM733771) (blue) [15,17]. (D) APA plots for H3K27ac HiCuT around pairs of H3K27ac-binding sites from NHEK cells (GSM733771) [15,17]. (E) WashU Epigenome browser view of two different genomic regions highlighting protein-directed chromatin interactions. The NHEK H3K27ac ChIP tracks are from ENCODE (blue) followed by location of SNPs associated with inflammatory skin diseases (red, NHGRI-EBI catalog, EFO_0000676) and chromatin interactions identified by H3K27ac HiCuT assay (red loops). Chr, Chromosome. (F) Comparison of H3K27ac-mediated long-range interactions in primary keratinocytes and GM12878 cells. The H3K27ac ChIP tracks are from ENCODE NHEK cells (GSM733771) followed by H3K27ac HiCuT interactions in primary keratinocytes or GM12878 cells. [15,17]
(TIF)

S1 Table Tables represent data metrics of HiCuT and SRA datasets. The valid interactions and unique valid interactions were obtained from HiC-Pro. The final column describes the number and percentage of interactions that fall within the respective ChIP peaks taken from ENCODE.
(XLSX)

S2 Table Table represents the HiCuT interactions generated in the study. The loop calls from the SRA dataset were taken from following sources: GM12878 HiChIP Smc1a: SE80820 GM12878. HiChIP CTCF: GSE115524 GM12878. HiC HICCUP loops: GSE63525 GM12878. ChIA-PET RNA Polymerase2 loops: GSM1872887.
(XLSX)

S3 Table Skin inflammatory disease specific SNPs and corresponding HiCuT interactions and genes. GWAS contains the GWAS dataset downloaded from NHGRI-EBI Catalog of human genome-wide association studies with accession number: EFO_0000676. SNP_matched_H3K27ac_interaction contains primary keratinocyte H3K27ac HiCuT interactions falling in the SNP region from GWAS. Genes_SNP_matched_interactions, contains the genes that were found overlapping in the regions from SNP_matched_H3K27ac_interaction.
(XLSX)

S4 Table Primer sequences. The i5 and i7 primer sequences used in HiCuT protocol.
(PDF)

S1 Text HiCuT Protocol. Step-by-step protocol.
(DOCX)

## References

[1] Bonev B, Cavalli G. Organization and function of the 3D genome. Nat Rev Genet. 2016;17(12):772. Epub 2017/07/14. doi: 10.1038/nrg.2016.147 . 28704353

[2] Sati S, Bonev B, Szabo Q, Jost D, Bensadoun P, Serra F, et al. 4D Genome Rewiring during Oncogene-Induced and Replicative Senescence. Mol Cell. 2020;78(3):522–38 e9. Epub 2020/03/30. doi: 10.1016/j.molcel.2020.03.007 ; PubMed Central PMCID: PMC7208559. 32220303 PMC7208559

[3] Lupianez DG, Kraft K, Heinrich V, Krawitz P, Brancati F, Klopocki E, et al. Disruptions of topological chromatin domains cause pathogenic rewiring of gene-enhancer interactions. Cell. 2015;161(5):1012–25. Epub 2015/05/12. doi: 10.1016/j.cell.2015.04.004 ; PubMed Central PMCID: PMC4791538. 25959774 PMC4791538

[4] Spielmann M, Lupianez DG, Mundlos S. Structural variation in the 3D genome. Nat Rev Genet. 2018;19(7):453–67. Epub 2018/04/26. doi: 10.1038/s41576-018-0007-0 . 29692413

[5] Fang R, Yu M, Li G, Chee S, Liu T, Schmitt AD, et al. Mapping of long-range chromatin interactions by proximity ligation-assisted ChIP-seq. Cell Res. 2016;26(12):1345–8. Epub 2016/11/26. doi: 10.1038/cr.2016.137 ; PubMed Central PMCID: PMC5143423. 27886167 PMC5143423

[6] Fullwood MJ, Liu MH, Pan YF, Liu J, Xu H, Mohamed YB, et al. An oestrogen-receptor-alpha-bound human chromatin interactome. Nature. 2009;462(7269):58–64. Epub 2009/11/06. doi: 10.1038/nature08497 ; PubMed Central PMCID: PMC2774924. 19890323 PMC2774924

[7] Mumbach MR, Rubin AJ, Flynn RA, Dai C, Khavari PA, Greenleaf WJ, et al. HiChIP: efficient and sensitive analysis of protein-directed genome architecture. Nat Methods. 2016;13(11):919–22. Epub 2016/11/01. doi: 10.1038/nmeth.3999 ; PubMed Central PMCID: PMC5501173. 27643841 PMC5501173

[8] Tang Z, Luo OJ, Li X, Zheng M, Zhu JJ, Szalaj P, et al. CTCF-Mediated Human 3D Genome Architecture Reveals Chromatin Topology for Transcription. Cell. 2015;163(7):1611–27. Epub 2015/12/22. doi: 10.1016/j.cell.2015.11.024 ; PubMed Central PMCID: PMC4734140. 26686651 PMC4734140

[9] Sati S, Cavalli G. Chromosome conformation capture technologies and their impact in understanding genome function. Chromosoma. 2017;126(1):33–44. Epub 2016/05/01. doi: 10.1007/s00412-016-0593-6 . 27130552

[10] Kaya-Okur HS, Wu SJ, Codomo CA, Pledger ES, Bryson TD, Henikoff JG, et al. CUT&Tag for efficient epigenomic profiling of small samples and single cells. Nat Commun. 2019;10(1):1930. Epub 2019/05/01. doi: 10.1038/s41467-019-09982-5 ; PubMed Central PMCID: PMC6488672. 31036827 PMC6488672

[11] Henikoff S, Henikoff JG, Kaya-Okur HS, Ahmad K. Efficient chromatin accessibility mapping in situ by nucleosome-tethered tagmentation. Elife. 2020;9. Epub 2020/11/17. doi: 10.7554/eLife.63274 ; PubMed Central PMCID: PMC7721439. 33191916 PMC7721439

[12] Lafontaine DL, Yang L, Dekker J, Gibcus JH. Hi-C 3.0: Improved Protocol for Genome-Wide Chromosome Conformation Capture. Curr Protoc. 2021;1(7):e198. Epub 2021/07/22. doi: 10.1002/cpz1.198 ; PubMed Central PMCID: PMC8362010. 34286910 PMC8362010

[13] Rao SS, Huntley MH, Durand NC, Stamenova EK, Bochkov ID, Robinson JT, et al. A 3D map of the human genome at kilobase resolution reveals principles of chromatin looping. Cell. 2014;159(7):1665–80. Epub 2014/12/17. doi: 10.1016/j.cell.2014.11.021 ; PubMed Central PMCID: PMC5635824. 25497547 PMC5635824

[14] Servant N, Varoquaux N, Lajoie BR, Viara E, Chen CJ, Vert JP, et al. HiC-Pro: an optimized and flexible pipeline for Hi-C data processing. Genome Biol. 2015;16:259. Epub 2015/12/02. doi: 10.1186/s13059-015-0831-x ; PubMed Central PMCID: PMC4665391. 26619908 PMC4665391

[15] Consortium EP. An integrated encyclopedia of DNA elements in the human genome. Nature. 2012;489(7414):57–74. Epub 2012/09/08. doi: 10.1038/nature11247 ; PubMed Central PMCID: PMC3439153. 22955616 PMC3439153

[16] Mumbach MR, Granja JM, Flynn RA, Roake CM, Satpathy AT, Rubin AJ, et al. HiChIRP reveals RNA-associated chromosome conformation. Nat Methods. 2019;16(6):489–92. Epub 2019/05/28. doi: 10.1038/s41592-019-0407-x ; PubMed Central PMCID: PMC6638558. 31133759 PMC6638558

[17] Pope BD, Ryba T, Dileep V, Yue F, Wu W, Denas O, et al. Topologically associating domains are stable units of replication-timing regulation. Nature. 2014;515(7527):402–5. Epub 2014/11/21. doi: 10.1038/nature13986 ; PubMed Central PMCID: PMC4251741. 25409831 PMC4251741

[18] Durand NC, Robinson JT, Shamim MS, Machol I, Mesirov JP, Lander ES, et al. Juicebox Provides a Visualization System for Hi-C Contact Maps with Unlimited Zoom. Cell Syst. 2016;3(1):99–101. Epub 2016/07/29. doi: 10.1016/j.cels.2015.07.012 ; PubMed Central PMCID: PMC5596920. 27467250 PMC5596920

[19] Chen EY, Tan CM, Kou Y, Duan Q, Wang Z, Meirelles GV, et al. Enrichr: interactive and collaborative HTML5 gene list enrichment analysis tool. BMC Bioinformatics. 2013;14:128. Epub 2013/04/17. doi: 10.1186/1471-2105-14-128 ; PubMed Central PMCID: PMC3637064. 23586463 PMC3637064

[20] Shi C, Ray-Jones H, Ding J, Duffus K, Fu Y, Gaddi VP, et al. Chromatin Looping Links Target Genes with Genetic Risk Loci for Dermatological Traits. J Invest Dermatol. 2021;141(8):1975–84. Epub 2021/02/20. doi: 10.1016/j.jid.2021.01.015 ; PubMed Central PMCID: PMC8315765. 33607115 PMC8315765

[21] Feng X, Zhou S, Cai W, Guo J. The miR-93-3p/ZFP36L1/ZFX axis regulates keratinocyte proliferation and migration during skin wound healing. Mol Ther Nucleic Acids. 2021;23:450–63. Epub 2021/01/22. doi: 10.1016/j.omtn.2020.11.017 ; PubMed Central PMCID: PMC7803633. 33473330 PMC7803633

[22] Wurm S, Zhang J, Guinea-Viniegra J, Garcia F, Munoz J, Bakiri L, et al. Terminal epidermal differentiation is regulated by the interaction of Fra-2/AP-1 with Ezh2 and ERK1/2. Genes Dev. 2015;29(2):144–56. Epub 2014/12/31. doi: 10.1101/gad.249748.114 ; PubMed Central PMCID: PMC4298134. 25547114 PMC4298134

[23] Mills AA, Zheng B, Wang XJ, Vogel H, Roop DR, Bradley A. p63 is a p53 homologue required for limb and epidermal morphogenesis. Nature. 1999;398(6729):708–13. Epub 1999/05/05. doi: 10.1038/19531 . 10227293

[24] Yang A, Kaghad M, Wang Y, Gillett E, Fleming MD, Dotsch V, et al. p63, a p53 homolog at 3q27-29, encodes multiple products with transactivating, death-inducing, and dominant-negative activities. Mol Cell. 1998;2(3):305–16. Epub 1998/10/17. doi: 10.1016/s1097-2765(00)80275-0 9774969

[25] Bhattacharyya S, Chandra V, Vijayanand P, Ay F. Identification of significant chromatin contacts from HiChIP data by FitHiChIP. Nat Commun. 2019;10(1):4221. Epub 2019/09/19. doi: 10.1038/s41467-019-11950-y ; PubMed Central PMCID: PMC6748947. 31530818 PMC6748947

[26] Akgol Oksuz B, Yang L, Abraham S, Venev SV, Krietenstein N, Parsi KM, et al. Systematic evaluation of chromosome conformation capture assays. Nat Methods. 2021;18(9):1046–55. Epub 2021/09/05. doi: 10.1038/s41592-021-01248-7 ; PubMed Central PMCID: PMC8446342. 34480151 PMC8446342

[27] Diaz N, Kruse K, Erdmann T, Staiger AM, Ott G, Lenz G, et al. Chromatin conformation analysis of primary patient tissue using a low input Hi-C method. Nat Commun. 2018;9(1):4938. Epub 2018/12/01. doi: 10.1038/s41467-018-06961-0 ; PubMed Central PMCID: PMC6265268. 30498195 PMC6265268

[28] Zhang C, Xu Z, Yang S, Sun G, Jia L, Zheng Z, et al. tagHi-C Reveals 3D Chromatin Architecture Dynamics during Mouse Hematopoiesis. Cell Rep. 2020;32(13):108206. Epub 2020/10/01. doi: 10.1016/j.celrep.2020.108206 . 32997998

[29] Nagano T, Lubling Y, Stevens TJ, Schoenfelder S, Yaffe E, Dean W, et al. Single-cell Hi-C reveals cell-to-cell variability in chromosome structure. Nature. 2013;502(7469):59–64. Epub 2013/09/27. doi: 10.1038/nature12593 ; PubMed Central PMCID: PMC3869051. 24067610 PMC3869051

[30] Ramani V, Deng X, Qiu R, Lee C, Disteche CM, Noble WS, et al. Sci-Hi-C: A single-cell Hi-C method for mapping 3D genome organization in large number of single cells. Methods. 2020;170:61–8. Epub 2019/09/20. doi: 10.1016/j.ymeth.2019.09.012 ; PubMed Central PMCID: PMC6949367. 31536770 PMC6949367

[31] Bartosovic M, Kabbe M, Castelo-Branco G. Single-cell CUT&Tag profiles histone modifications and transcription factors in complex tissues. Nat Biotechnol. 2021;39(7):825–35. Epub 2021/04/14. doi: 10.1038/s41587-021-00869-9 ; PubMed Central PMCID: PMC7611252. 33846645 PMC7611252

[32] Wu SJ, Furlan SN, Mihalas AB, Kaya-Okur HS, Feroze AH, Emerson SN, et al. Single-cell CUT&Tag analysis of chromatin modifications in differentiation and tumor progression. Nat Biotechnol. 2021;39(7):819–24. Epub 2021/04/14. doi: 10.1038/s41587-021-00865-z ; PubMed Central PMCID: PMC8277750. 33846646 PMC8277750

[33] Zhang Y, Liu T, Meyer CA, Eeckhoute J, Johnson DS, Bernstein BE, et al. Model-based analysis of ChIP-Seq (MACS). Genome Biol. 2008;9(9):R137. Epub 2008/09/19. doi: 10.1186/gb-2008-9-9-r137 ; PubMed Central PMCID: PMC2592715. 18798982 PMC2592715

[34] Ramirez F, Ryan DP, Gruning B, Bhardwaj V, Kilpert F, Richter AS, et al. deepTools2: a next generation web server for deep-sequencing data analysis. Nucleic Acids Res. 2016;44(W1):W160–5. Epub 2016/04/16. doi: 10.1093/nar/gkw257 ; PubMed Central PMCID: PMC4987876. 27079975 PMC4987876

[35] Dekker J, Belmont AS, Guttman M, Leshyk VO, Lis JT, Lomvardas S, et al. The 4D nucleome project. Nature. 2017;549(7671):219–26. Epub 2017/09/15. doi: 10.1038/nature23884 ; PubMed Central PMCID: PMC5617335. 28905911 PMC5617335

[36] Zhou X, Maricque B, Xie M, Li D, Sundaram V, Martin EA, et al. The Human Epigenome Browser at Washington University. Nat Methods. 2011;8(12):989–90. Epub 2011/12/01. doi: 10.1038/nmeth.1772 ; PubMed Central PMCID: PMC3552640. 22127213 PMC3552640

[37] Heinz S, Benner C, Spann N, Bertolino E, Lin YC, Laslo P, et al. Simple combinations of lineage-determining transcription factors prime cis-regulatory elements required for macrophage and B cell identities. Mol Cell. 2010;38(4):576–89. Epub 2010/06/02. doi: 10.1016/j.molcel.2010.05.004 ; PubMed Central PMCID: PMC2898526. 20513432 PMC2898526

[38] Durand NC, Shamim MS, Machol I, Rao SS, Huntley MH, Lander ES, et al. Juicer Provides a One-Click System for Analyzing Loop-Resolution Hi-C Experiments. Cell Syst. 2016;3(1):95–8. Epub 2016/07/29. doi: 10.1016/j.cels.2016.07.002 ; PubMed Central PMCID: PMC5846465. 27467249 PMC5846465

