# Supplementary figures and images for: HiCuT: An efficient and low input method to identify protein-directed chromatin interactions
Source: PLoS Genet. 2022 Mar 23;18(3):e1010121. doi: 10.1371/journal.pgen.1010121 (PMC8979432; doi:10.1371/journal.pgen.1010121)

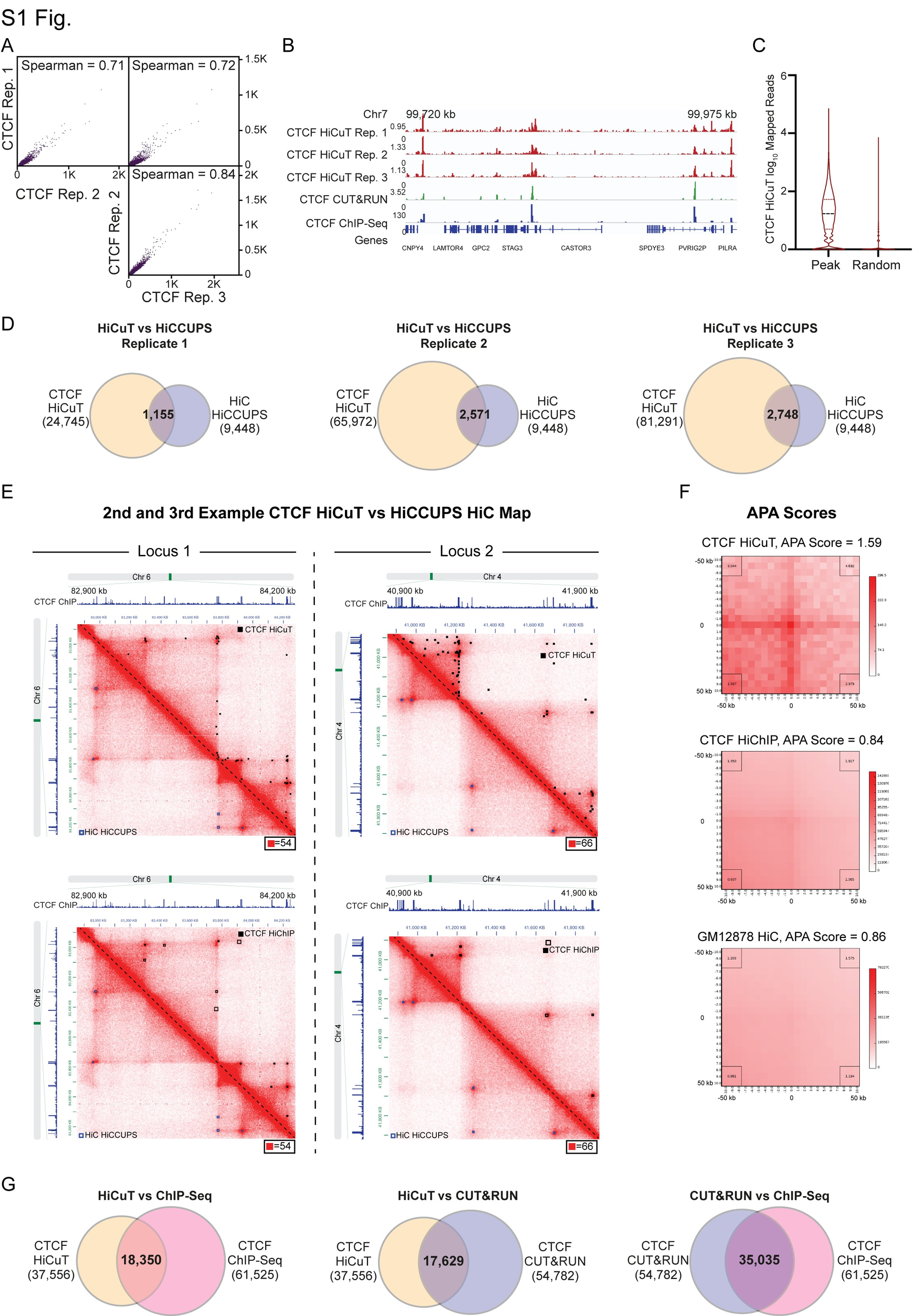

Supplement: S1 Fig — (A) Scatter Plot correlation of aligned reads from HiCuT replicates. Spearman r is indicated. (B) Genome browser snapshot showing GM12878 CTCF HiCuT tracks (red), CTCF CUT&RUN Tracks from 4DN Network (green, 4DNES6GVE8XZ) and CTCF ChIP-Seq tracks from ENCODE (blue, GSM733752) [15,17,35]. (C) Violin plots of mapped reads from CTCF HiCuT datasets at CTCF ChIP peaks and random sites. (D) Comparison of HiCuT shared long-range chromatin interactions between Hi-C HICCUPS loop reference data set and HiCuT replicates. (E) In situ GM12878 Hi-C contact map of two regions at 5 kb resolution, superimposed with HiCuT interactions (top panels, upper right, black boxes), GM12878 Hi-C HiCCUPS loops (all panels, lower left, open blue boxes) and GM12878 CTCF HiChIP loops (lower panels, upper right, black boxes). Maximum intensity is indicated in the lower right of each panel. (F) APA plots from CTCF HiCuT, CTCF HiChIP and GM12878 HiC, around pairs of CTCF-binding sites from GM12878 cells. (G) Comparison of CTCF peaks obtained from GM12878 cells using HiCuT, ChIP-Seq (ENCODE GSM733752) and CUT&RUN (4DN network 4DNES6GVE8XZ). Number of identified peaks are shown. (TIF) [file pgen.1010121.s001.tif]

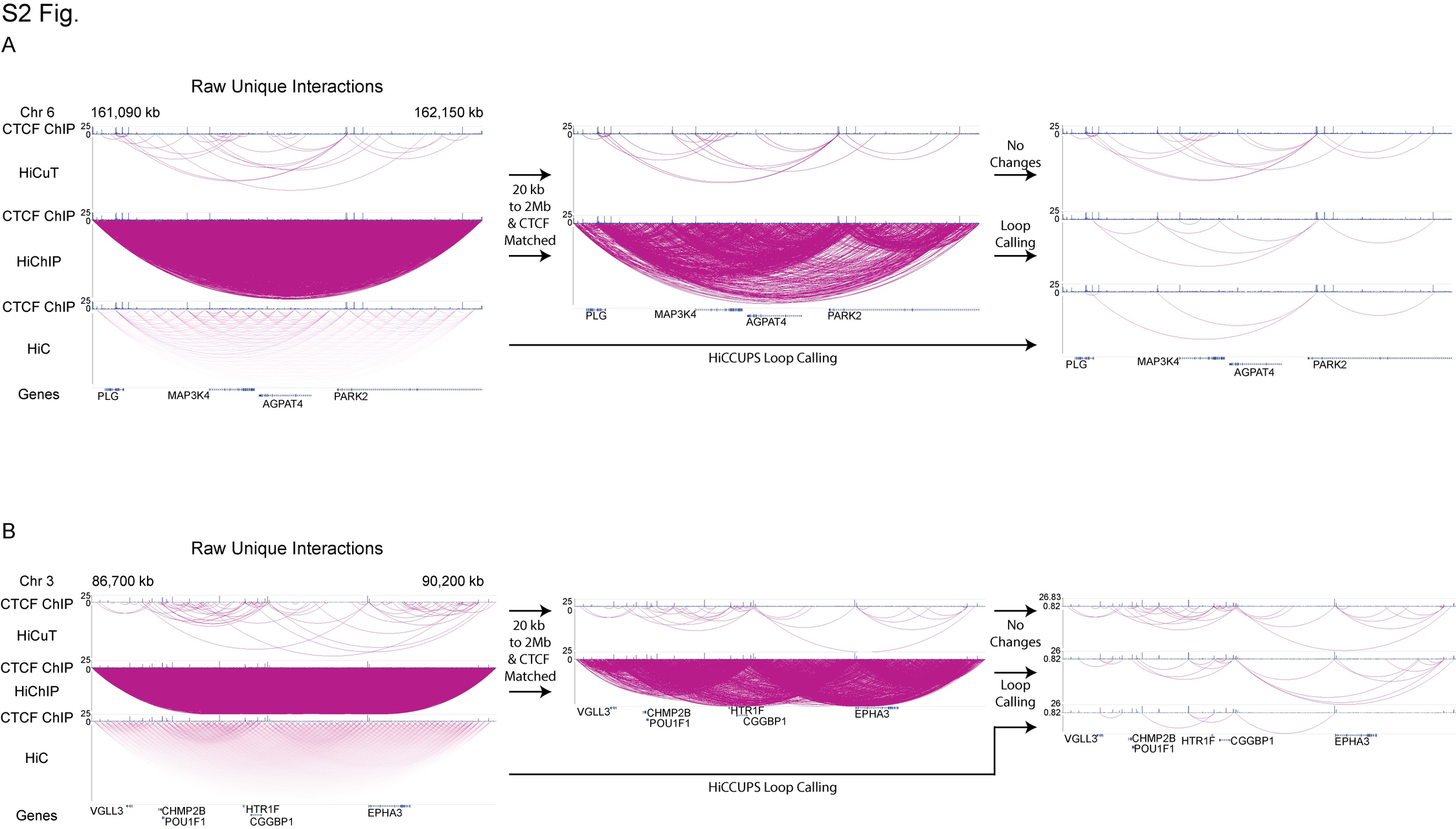

Supplement: S2 Fig — (A) and (B). Two different chromosome regions are displayed. The left panel displays raw unique interactions for HiCuT, HiChIP, and Hi-C in GM12878 cells. The HiC interactions are taken from WashU browser (25 Kb bin, KR Norm). The middle panel displays filtered unique interactions for HiCuT and HiChIP. Captured interactions were between 20Kb– 2Mb in length, with at least one anchor overlapping with a known CTCF ChIP-seq peak. The right panel displays final long-range interactions after looping calling programs were performed for HiChIP and HiC datasets. HiCuT did not require additional filtering. ChIP-Seq tracks are obtained from ENCODE GM12878 dataset (GSM733752), and gene names are listed below [15,17]. Chr, Chromosome (TIF) [file pgen.1010121.s002.tif]

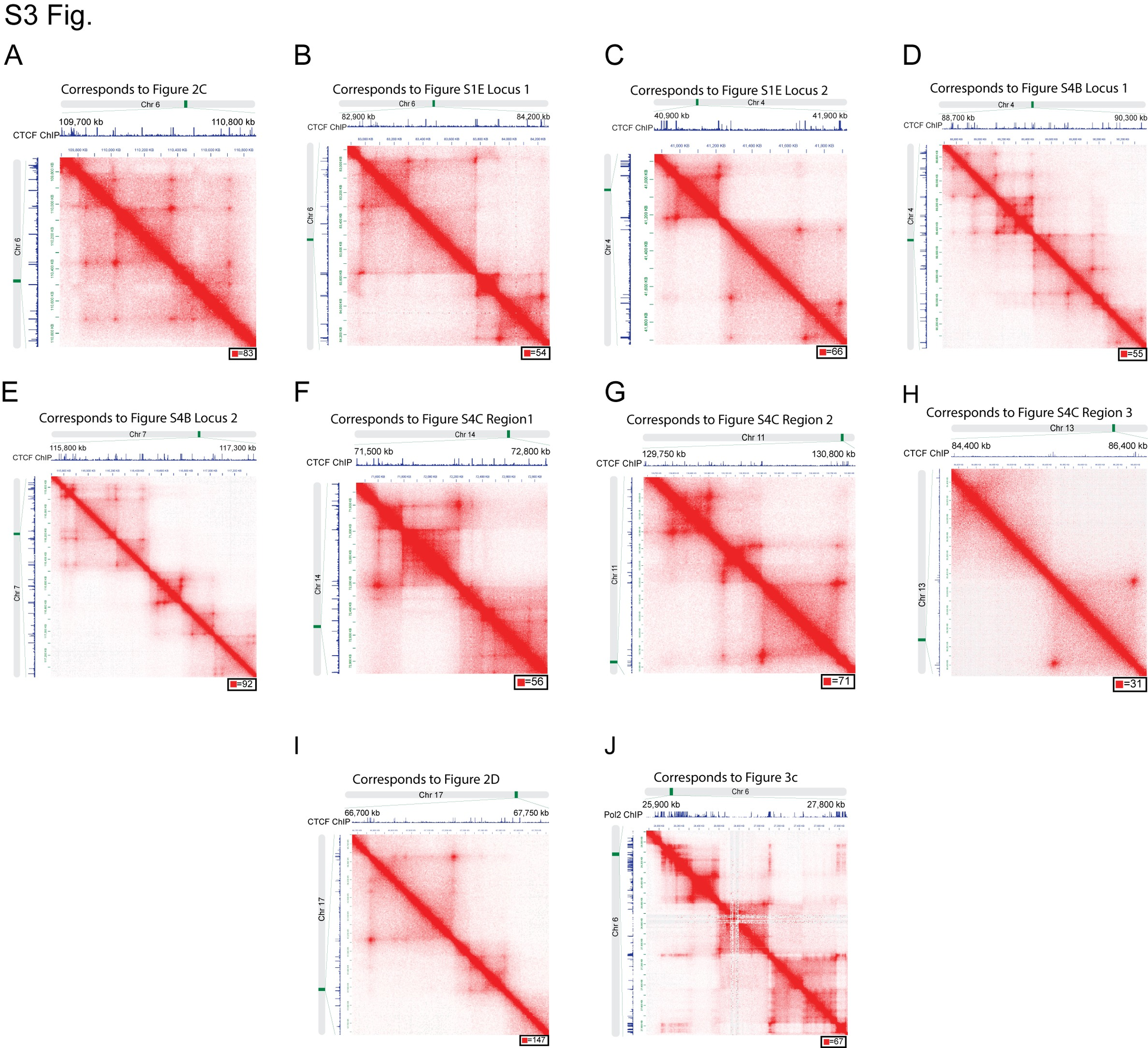

Supplement: S3 Fig — In situ GM12878 Hi-C data maps generated from juicer [13,18,38]. No datasets are superimposed. The corresponding figure panel is indicated at the top of each map. (TIF) [file pgen.1010121.s003.tif]

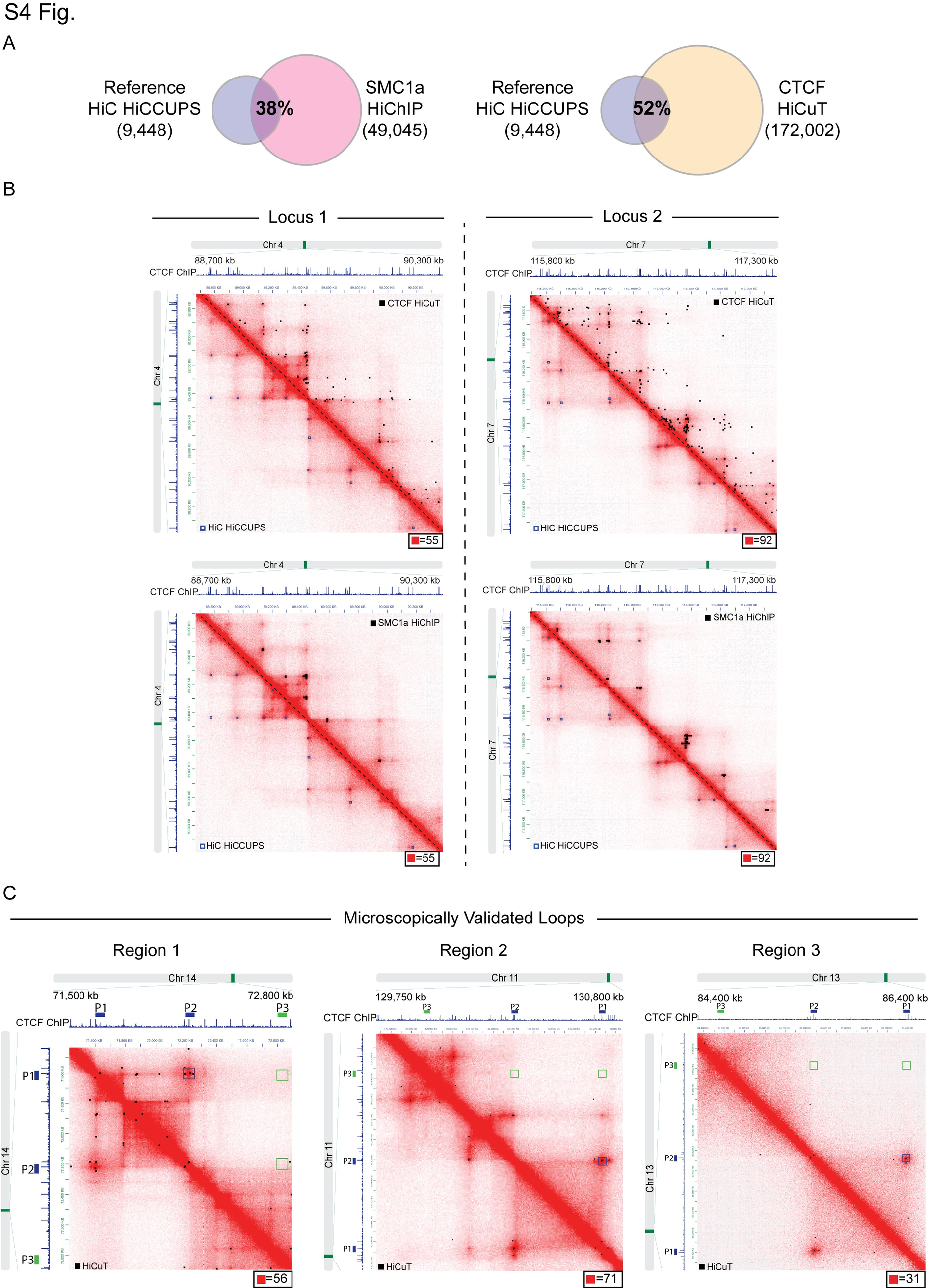

Supplement: S4 Fig — (A) Comparison of shared long-range chromatin interactions between Hi-C reference data set, GM12878 CTCF HiCuT, and GM12878 SMC1a HiChIP. The number of interactions for each dataset are displayed [7]. (B) GM12878 Hi-C contact maps at two different loci. Hi-C dataset at 5kb resolution superimposed with HiCuT interactions (top panels, upper right, black boxes), GM12878 Hi-C HiCCUPS loops (all panels, lower left, open blue boxes) and GM12878 SMC1a HiChIP loops (lower panels, upper right, black boxes) (GEO GSE80820). Maximum intensity is indicated in the lower right of each panel [7]. (C) HiCuT captures previously published microscopically validated loops [13]. GM12878 Hi-C contact map with superimposed location of different DNA FISH probes. The blue probes (P1 and P2) were shown to interact in a DNA FISH experiment (blue rectangle), and HiCuT detected this interaction [13]. The green boxes represent non interacting regions between FISH probes (P1 to P3, green boxes). GM12878 CTCF Hi-CuT interactions (black boxes) superimposed on the in situ Hi-C map. Maximum intensity is indicated in the lower right of each panel. (TIF) [file pgen.1010121.s004.tif]

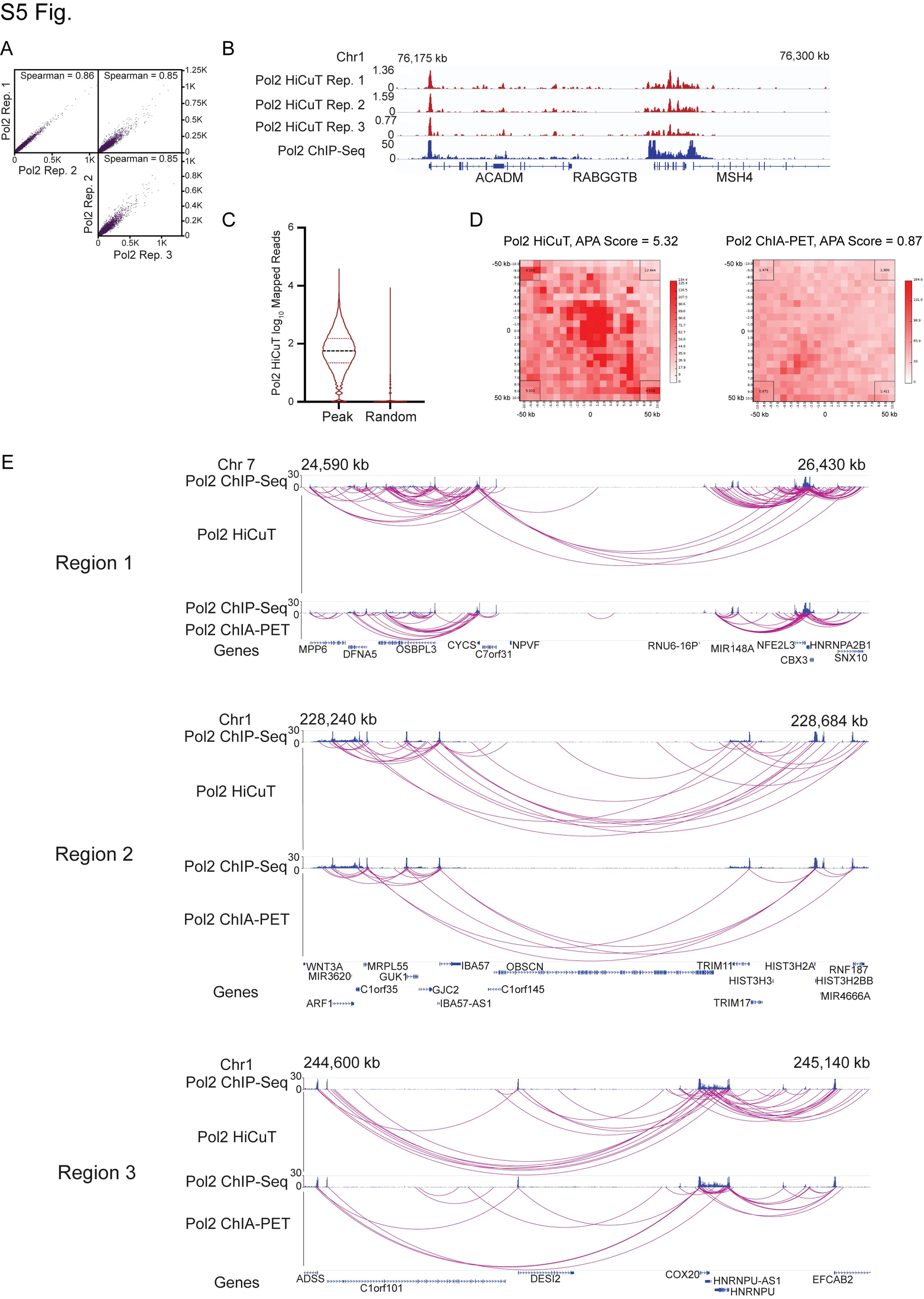

Supplement: S5 Fig — (A) Scatter plot correlation of aligned reads from HiCuT replicates. Spearman r is indicated. (B) Genome browser snapshot showing GM12878 RNA polymerase 2 HiCuT tracks (red) and RNA polymerase 2 ChIP-Seq tracks from ENCODE (blue, GSM935386) [15,17]. (C) Violin plot of mapped reads from HiCuT datasets at RNA polymerase 2 ChIP peaks and random sites. (D) APA plots for Pol2 HiCuT and Pol2 ChIA-PET around pairs of Pol2-binding sites from GM12878 cells. (E) WashU epigenome browser view of three different genomic regions highlighting protein-directed chromatin interactions. The RNA polymerase 2 ChIP tracks are from ENCODE GM12878 cells (GSM935386), followed by chromatin interactions identified by HiCuT and ChIA-PET assays [15,17]. Chr, Chromosome. (TIF) [file pgen.1010121.s005.tif]

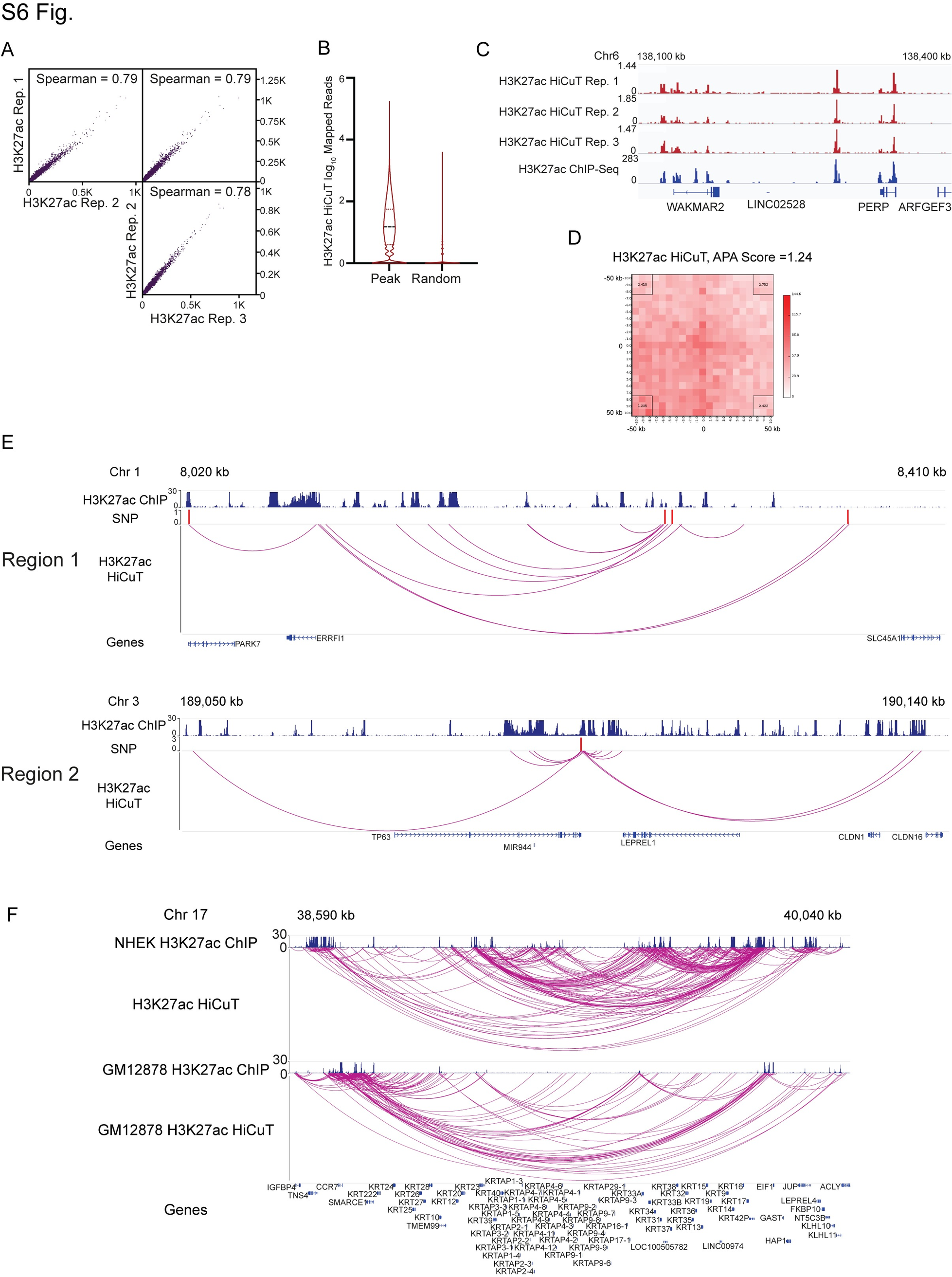

Supplement: S6 Fig — (A) Scatter plot correlation of aligned reads from HiCuT replicates. Spearman r is indicated. (B) Violin plot of mapped reads from HiCuT datasets at H3K27ac ChIP peaks and random sites. (C) Genome browser snapshot showing primary keratinocyte H3K27ac HiCuT tracks (red) and NHEK H3K27ac ChIP-Seq tracks ENCODE (GSM733771) (blue) [15,17]. (D) APA plots for H3K27ac HiCuT around pairs of H3K27ac-binding sites from NHEK cells (GSM733771) [15,17]. (E) WashU Epigenome browser view of two different genomic regions highlighting protein-directed chromatin interactions. The NHEK H3K27ac ChIP tracks are from ENCODE (blue) followed by location of SNPs associated with inflammatory skin diseases (red, NHGRI-EBI catalog, EFO_0000676) and chromatin interactions identified by H3K27ac HiCuT assay (red loops). Chr, Chromosome. (F) Comparison of H3K27ac-mediated long-range interactions in primary keratinocytes and GM12878 cells. The H3K27ac ChIP tracks are from ENCODE NHEK cells (GSM733771) followed by H3K27ac HiCuT interactions in primary keratinocytes or GM12878 cells. [15,17] (TIF) [file pgen.1010121.s006.tif]
