# Supplementary material for: HiCuT: An efficient and low input method to identify protein-directed chromatin interactions
Source: PLoS Genet. 2022 Mar 23;18(3):e1010121. doi: 10.1371/journal.pgen.1010121 (PMC8979432; doi:10.1371/journal.pgen.1010121)
Supplement: S4 Table — The i5 and i7 primer sequences used in HiCuT protocol. (PDF) [file pgen.1010121.s010.pdf]

Supplementary Table S4

Primer Sequences

Name	Sequence	Barcode
Universal i5	AATGATACGGCGACCACCGAGATCTACACTAGATCGCTCGTCGGCAGCGTCAGATGTGTAT	
i7_1	CAAGCAGAAGACGGCATACGAGATTCGCCTTAGTCTCGTGGGCTCGGAGATGTG	TCGCCTTA
i7_2	CAAGCAGAAGACGGCATACGAGATCTAGTACGGTCTCGTGGGCTCGGAGATGTG	CTAGTACG
i7_3	CAAGCAGAAGACGGCATACGAGATTTCTGCCTGTCTCGTGGGCTCGGAGATGTG	TTCTGCCT
i7_4	CAAGCAGAAGACGGCATACGAGATGCTCAGGAGTCTCGTGGGCTCGGAGATGTG	GCTCAGGA
i7_5	CAAGCAGAAGACGGCATACGAGATAGGAGTCCGTCTCGTGGGCTCGGAGATGTG	AGGAGTCC
i7_6	CAAGCAGAAGACGGCATACGAGATCATGCCTAGTCTCGTGGGCTCGGAGATGTG	CATGCCTA
i7_7	CAAGCAGAAGACGGCATACGAGATGTAGAGAGGTCTCGTGGGCTCGGAGATGTG	GTAGAGAG
i7_8	CAAGCAGAAGACGGCATACGAGATCCTCTCTGGTCTCGTGGGCTCGGAGATGTG	CCTCTCTG
i7_9	CAAGCAGAAGACGGCATACGAGATAGCGTAGCGTCTCGTGGGCTCGGAGATGTG	AGCGTAGC
i7_10	CAAGCAGAAGACGGCATACGAGATCAGCCTCGGTCTCGTGGGCTCGGAGATGTG	CAGCCTCG
i7_11	CAAGCAGAAGACGGCATACGAGATTGCCTCTTGTCTCGTGGGCTCGGAGATGTG	TGCCTCTT
i7_12	CAAGCAGAAGACGGCATACGAGATTCCTCTACGTCTCGTGGGCTCGGAGATGTG	TCCTCTAC
i7_11	CAAGCAGAAGACGGCATACGAGATTGCCTCTTGTCTCGTGGGCTCGGAGATGTG	TGCCTCTT
i7_12	CAAGCAGAAGACGGCATACGAGATTCCTCTACGTCTCGTGGGCTCGGAGATGTG	TCCTCTAC
