# Supplementary material for: HiCuT: An efficient and low input method to identify protein-directed chromatin interactions
Source: PLoS Genet. 2022 Mar 23;18(3):e1010121. doi: 10.1371/journal.pgen.1010121 (PMC8979432; doi:10.1371/journal.pgen.1010121)
Supplement: S1 Text — Step-by-step protocol. (DOCX) [file pgen.1010121.s011.docx]

**Step by step HiCuT Protocol**

**Cell lysis and nuclei fixation**

1. Fix 100,000 cells in 0.5 mL of freshly made 1% formaldehyde solution at room temperature for 10 minutes.
2. Add glycine (200 mM final) and incubate it for 5 minutes at room temperature and then 15 minutes on ice.
3. Spin down at 2,000 g for 5 minutes and wash the cells with 0.5% BSA - PBS.
4. Remove the supernatant and resuspend the pellet in 3mM DSG (final concentration) in 0.5 mL PBS.
5. Incubate at room temperature for 40 minutes, on rotation.
6. Add glycine (400 mM final concentration) and incubate samples for 5 minutes at room temperature.
7. Spin down cells at 2,000 g for 5 minutes and wash with 0.5% BSA - PBS.
8. Resuspend cells in Hi-C lysis buffer and incubate on ice for 30 minutes.
9. Spin down the cells at 2,500 g for 5 minutes and wash the nuclei once with NEBuffer 3.1.

**In situ contact generation**

1. Resuspend the nuclei pellet in 161 μL of 1x NEBuffer 3.1.
2. Permeabilize nuclei by adding 19 μL of 1% SDS and incubate for 10 minutes at 65 °C without shaking.
3. Immediately place the tube on ice after incubation.
4. Quench SDS by adding 21.5 μL of 10% Triton X-100 and incubate the samples at 37 °C for 15 minutes with shaking at 900 rpm.
5. Add 20 μL of 10U/μL DdeI, 4 μL of 50U/μL DpnII, and 2 μL of 1x NEBuffer 3.1 and mix gently by pipetting.
6. Incubate the mixture for 3 hours or overnight at 37 °C on a thermomixer at 900 rpm, in 30 seconds on, 4 minutes off mode.
7. After digestion, inactivate the enzymes at 65 °C for 20 minutes without shaking.
8. To fill in restriction fragment overhangs, add 35 μL of end-filling master mix:

18.75 μL of 0.4 mM dATP

0.75 μL of 10mM dTTP

0.75 μL of 10mM dGTP

0.75 μL of 10mM dCTP

3.5 μL of 10x NEBuffer 3.1

5.5 μL of water

5 μL of DNA polymerase I (NEB, M0210).

1. Keep samples on rotation for 2-3 hours at 37 °C.
2. Ligate the DNA fragments by adding 332.5 μL of ligation master mix containing:

60 μL of 10X NEB T4 DNA ligase buffer (NEB, B0202),

50 μL of 10% Triton X-100,

6 μL of 10 mg/mL BSA,

2.5 μL of 400 U/μL T4 DNA Ligase (NEB, M0202),

214 μL of water.

1. Keep samples on rotation for 2-3 hours at room temperature.
2. Spin down the nuclei at 3,500 g for 5 minutes and wash the nuclei once with 200 μL of exchange buffer.
3. Resuspend the proximity ligated nuclei in 100 μL of exchange buffer and keep them on ice.

**Chromatin cleavage and tagmentation**

1. Wash 11 μL/sample of Concanavalin-A Beads two times with 100 μL of bead activation buffer.
2. Resuspend activated beads in 10 μL exchange buffer.
3. Add and mix 10 μL of activated beads to 100 μL of exchange buffer containing proximity ligated nuclei.
4. Incubate the reaction mix for 10 minutes at room temperature.
5. Place tubes on magnet and remove the supernatant.
6. Add 50 μL of cold antibody buffer and 1 ug of the appropriate primary antibody.
7. Incubate samples for 2 hours at room temperature or overnight at 4 °C on a rotating platform.
8. Place the samples on a magnetic stand and remove the supernatant. Add 50 μL of cold low-salt digitonin buffer and 0.25 ug of secondary antibody.
9. Incubate samples at room temperature for 30 minutes.
10. Wash twice with 200 μL of cold low salt digitonin buffer.
11. Add 50 μL of ice-cold high salt digitonin buffer and 2.5 μL of CUTANA pAG-Tn5 (20x stock from EpiCypher) and incubate at room temperature for 1 hour.
12. Wash samples twice with 200 μL cold high salt digitonin buffer.
13. Resuspend beads in 50 μL of cold tagmentation buffer and incubate at 37 °C for 1 hour.
14. Remove the supernatant and resuspend the samples in 50 μL of Release Buffer.
15. Incubate samples at 58°C for 1 hour and 68°C for 2 hours in a thermocycler.
16. After incubation, collect the supernatant and purify with Zymo Research ChIP DNA Clean & Concentrator protocol (Cat No. D5210), as per manufacturer’s recommendation. Elute the PCR-ready HiCuT libraries in 21 μL volume.

**Buffers**

Hi-C lysis buffer

10mM Tris-HCl pH8.0

10mM NaCl

0.2% Igepal CA630,

1X protease inhibitor

Exchange buffer

20 mM HEPES-KOH pH 7.9

10 mM KCl,

0.1% Triton X-100

20% Glycerol

0.5 mM Spermidine

1x EDTA-free Protease Inhibitor

Bead activation buffer

20 mM HEPES, pH 7.9

10 mM KCl

1 mM CaCl2

1 mM MnCl2

Antibody buffer

20 mM HEPES pH 7.5, 150 mM NaCl

0.5 mM Spermidine

1x EDTA-free Protease Inhibitor,

0.01% Digitonin

2 mM EDTA

Low-salt digitonin buffer

20 mM HEPES pH 7.5

150 mM NaCl

0.5 mM Spermidine

1x EDTA-free Protease Inhibitor

0.01% Digitonin

High salt digitonin buffer

20 mM HEPES pH 7.5

300 mM NaCl

0.5 mM Spermidine

1x EDTA-free Protease Inhibitor

0.01% Digitonin

Tagmentation buffer

20 mM HEPES pH 7.5

300 mM NaCl

0.5 mM Spermidine

1x EDTA-free Protease Inhibitor

10 mM MgCl2

Release Buffer

10 mM TAPS pH 8.5

0.5% SDS

22.5mM EDTA

1uL of 10mg/mL Proteinase K
